# Supplementary material for: Health selection on self-rated health and the healthy migrant effect: Baseline and 1-year results from the health of Philippine Emigrants Study
Source: PLOS Glob Public Health. 2022 Jul 22;2(7):e0000324. doi: 10.1371/journal.pgph.0000324 (PMC9450558; doi:10.1371/journal.pgph.0000324)
Supplement: S1 Table — (DOCX) [file pgph.0000324.s001.docx]

**S1 Table.** Allostatic load components and clinical risk cutoffs

| **Biomarker** | **Cut Point for Clinical Risk** | **Source for Clinical Risk** |
| --- | --- | --- |
| Systolic Blood Pressure | >130 mmHg | Whelton et al. 2018[1] |
| Diastolic Blood Pressure | >80 mmHg | Whelton et al. 2018[1] |
| Body Mass Index | >27.5 kg/m^2^ | World Health Organization[2] |
| Waist Circumference | Men: >= 102 cm  Women: >= 88 cm | World Health Organization[3] |
| Waist-to-Hip Ratio | Men: >=.90 cm  Women: >= .85cm | World Health Organization[3] |
| Total Cholesterol | >=240 mg/dL | Ma & Shieh 2006[4] |
| Low Density Lipoprotein | >= 160 mg/dL | Ma & Shieh 2006[4] |
| High Density Lipoprotein | Men: < 40mg/dL  Women: < 50 mg/dL | Ma & Shieh 2006[4] |
| Triglycerides | >= 200 mg/dL | Ma & Shieh 2006[4] |
| C-Reactive Protein | >10 mg/L | Nehring et al. 2020[5] |
| Apolipoprotein-B | >120 mg/dL | Cao et al. 2018[6] |

**References**

1. Whelton PK, Carey RM, Aronow WS, Casey DE, Collins KJ, Dennison Himmelfarb C, et al. 2017 ACC/AHA/AAPA/ABC/ACPM/AGS/APhA/ASH/ASPC/NMA/PCNA Guideline for the Prevention, Detection, Evaluation, and Management of High Blood Pressure in Adults. Journal of the American College of Cardiology. 2018;71(19):e127. doi: 10.1016/j.jacc.2017.11.006.

2. World Health Organization. Obesity: preventing and managing the global epidemic: World Health Organization; 2000.

3. World Health Organization. Waist Circumference and Waist-Hip Ratio. Geneva, Switzerland: World Health Organization, 2008 December 8, 2008. Report No.

4. Ma H, Shieh K-J. Cholesterol and human health. The Journal of American Science. 2006;2(1):46-50.

5. Nehring S, Goyal A, Bansal P, Patel B. C Reactive Protein (CRP). StatPearls. Treasure Island, FL: StatPearls Publishing; 2020.

6. Cao J, Steffen BT, Guan W, Remaley AT, McConnell JP, Palamalai V, et al. A comparison of three apolipoprotein B methods and their associations with incident coronary heart disease risk over a 12-year follow-up period: The Multi-Ethnic Study of Atherosclerosis. Journal of Clinical Lipidology. 2018;12(2):300-4. doi: <https://doi.org/10.1016/j.jacl.2017.12.013>.
