# Supplementary material for: Health selection on self-rated health and the healthy migrant effect: Baseline and 1-year results from the health of Philippine Emigrants Study
Source: PLOS Glob Public Health. 2022 Jul 22;2(7):e0000324. doi: 10.1371/journal.pgph.0000324 (PMC9450558; doi:10.1371/journal.pgph.0000324)
Supplement: S2 Table — (DOCX) [file pgph.0000324.s002.docx]

**S2 Table.** Weighted means and proportions of covariates by inclusion in the 1-year sample (vs. full sample), Health of Philippine Emigrants Study (HoPES), N = 1637

| Characteristic | Not in Analytical Sample (n=443) | | | | In Analytical Sample  (n=1194) | | | |  |  | |  | |
| --- | --- | --- | --- | --- | --- | --- | --- | --- | --- | --- | --- | --- | --- |
|  | Total  (n=443) | Non-Migrant  (n = 51) | Migrant  (n=392) | Total  (n=1194) | | Non-Migrant  (n=754) | Migrant  (n=440) | p-value between in sample vs. not | | | p-value between non-migrants in sample vs. not | | p-value between migrants in sample vs. not |
| **Self-Rated Health** |  |  |  |  | |  |  | < .001 | | | .791 | | .004 |
| % Excellent | 7.8 | 2.7 | 8.4 | 5.9 | | 3.1 | 10.6 |  | | |  | |  |
| % Very Good | 20.0 | 9.1 | 21.4 | 13.7 | | 6.0 | 26.9 |  |  |  |  | |  |
| % Good | 35.8 | 25.6 | 37.1 | 31.6 | | 26.2 | 41.0 |  |  |  |  | |  |
| % Fair | 33.5 | 48.6 | 31.6 | 42.1 | | 54.9 | 19.9 |  |  |  |  | |  |
| %Poor | 2.9 | 14.0 | 1.5 | 6.8 | | 9.7 | 1.6 |  |  |  |  | |  |
| **Mean Self- Rated Health (SE) (0-4)** | 1.96 (0.05) | 1.38 (0.14) | 2.04 (0.05) | 1.70 (0.03) | | 1.38 (0.03) | 2.25 (0.05) | < .001 | | | .995 | | .001 |
| **Demographic Factors** |  |  |  |  | |  |  |  | | |  | |  |
| Mean Age (SE) | 38.14 (0.57) | 35.35 (1.56) | 38.50 (0.62) | 36.49 (0.33) | | 37.11 (0.43) | 35.43 (0.53) | .013 | | | .274 | | < .001 |
| % Male | 36.6 | 34.4 | 36.9 | 32.4 | | 33.5 | 30.6 | .123 | | | .908 | | .058 |
| % Any English | 12.3 | 10.8 | 12.5 | 7.5 | | 4.1 | 13.4 | .002 | | | .033 | | .696 |
| Educational Attainment |  |  |  |  | |  |  | < .001 | | | .242 | | < .001 |
| % Less than high school | 11.5 | 10.8 | 11.6 | 9.9 | | 12.8 | 4.8 |  | | |  | |  |
| % High school graduate | 23.4 | 16.8 | 24.2 | 16.7 | | 16.1 | 17.8 |  |  |  |  | |  |
| % Some college | 20.5 | 25.9 | 19.9 | 30.3 | | 38.2 | 16.8 |  |  |  |  | |  |
| % College degree and above | 44.6 | 46.4 | 44.4 | 43.1 | | 32.9 | 60.7 |  |  |  |  | |  |
| Financial Strain ^1^ |  |  |  |  | |  |  | .001 | | | .536 | | .067 |
| % High | 23.4 | 50.4 | 20.0 | 32.5 | | 42.4 | 15.4 |  | | |  | |  |
| % Medium | 54.4 | 39.4 | 56.4 | 50.1 | | 47.4 | 54.8 |  |  |  |  | |  |
| % Low | 22.2 | 10.2 | 23.7 | 17.4 | | 10.3 | 29.9 |  |  |  |  | |  |
| Healthcare Utilization |  |  |  |  | |  |  | .006 | | | .169 | | .737 |
| % No Treatment | 46.1 | 23.9 | 49.0 | 40.7 | | 34.9 | 50..6 |  | | |  | |  |
| % Hospital | 34.8 | 46.2 | 33.3 | 32.5 | | 33.5 | 30.7 |  |  |  |  | |  |
| % Clinic and Other | 19.2 | 29.9 | 17.8 | 26.9 | | 31.6 | 18.7 |  |  |  |  | |  |

^1^ High financial strain indicates that participants had “Some to considerable difficulty in meeting expenses”. Medium financial strain indicates that participants had “just enough to pay expenses without difficulty”. Low financial strain indicates that participants had “enough money with money leftover”.
