# Supplementary material for: Health selection on self-rated health and the healthy migrant effect: Baseline and 1-year results from the health of Philippine Emigrants Study
Source: PLOS Glob Public Health. 2022 Jul 22;2(7):e0000324. doi: 10.1371/journal.pgph.0000324 (PMC9450558; doi:10.1371/journal.pgph.0000324)
Supplement: S5 Table — (DOCX) [file pgph.0000324.s005.docx]

**S5 Table.** Binary logistic regression of 1-year good/very good/excellent self-rated health on migrant status, Health of Philippine Emigrants Study (HoPES), N = 1194

|  | Full Model | |
| --- | --- | --- |
| Variables | OR | 95% CI |
| **Migrant Status (Non-migrant ref.)** | 2.11 | 1.33, 3.35 |
| **Demographic Domain** |  |  |
| Age | 0.99 | 0.97, 1.00 |
| Gender (Female ref.)^1^ |  |  |
| Male | 0.92 | 0.67. 1.27 |
| Interview Language (No English ref.) |  |  |
| Any English | 1.44 | 0.90, 2.30 |
| **Physical Health** |  |  |
| Allostatic load^1^ | 0.93 | 0.86, 1.01 |
| **Mental Health** |  |  |
| Emotional distress | 0.77 | 0.60, 0.98 |
| Perceived stress | 0.78 | 0.58, 1.04 |
| Cognitive functioning^1,2^ | 0.96 | 0.92, 1.01 |
| **Health Behavior Domain** |  |  |
| Physical Activity (Low ref.) |  |  |
| High physical activity | 1.38 | 0.96, 1.98 |
| Fast Food consumption (High ref.) |  |  |
| Low fast food consumption | 0.93 | 0.69, 1.26 |
| Soda Consumption (High ref.) |  |  |
| Low soda consumption | 1.15 | 0.84, 1.58 |
| Vegetable Consumption (Low ref.) |  |  |
| High vegetable consumption | 1.19 | 0.78, 1.84 |
| Fruit Consumption (Low ref.) |  |  |
| High fruit consumption | 0.79 | 0.49, 1.26 |
| Hours of Sleep (7-to-9 hours ref.) |  |  |
| Less than 7 hours | 1.14 | 0.80, 1.62 |
| More than 9 hours | 0.93 | 0.59, 1.47 |
| Sleep Quality | 2.54 | 2.08, 3.11 |
| **Socioeconomic and Healthcare Utilization Domain** |  |  |
| Educational Attainment (less than high school ref.)^1^ |  |  |
| High school graduate | 0.94 | 0.53, 1.66 |
| Some college | 1.27 | 0.74, 2.16 |
| College degree and above | 0.97 | 0.57, 1.65 |
| Financial Strain (High ref.)^3^ |  |  |
| Medium | 1.61 | 1.11, 2.33 |
| Low | 2.70 | 1.70, 4.28 |
| Healthcare Utilization (No treatment ref.)^1^ |  |  |
| Hospital | 1.28 | 0.91, 1.81 |
| Clinic or other | 0.98 | 0.68, 1.42 |
| **Social Capital** |  |  |
| Social Capital | 1.03 | 0.98, 1.09 |
| Social isolation (Low ref.) |  |  |
| High social isolation | 1.19 | 0.42, 3.32 |
| **Social Desirability (Low ref.)^1, 4^** |  |  |
| High social desirability | 0.55 | 0.19, 1.60 |
| Constant | 0.13 | 0.03, 0.51 |

^1^ Indicates variable measured at baseline only.

^2^ Higher cognitive functioning score indicates worse cognitive functioning.

^3^ High financial strain indicates that participants had “Some to considerable difficulty in meeting expenses”. Medium financial strain indicates that participants had “just enough to pay expenses without difficulty”. Low financial strain indicates that participants had “enough money with money leftover”.

^4^ High social desirability refers to people who “sometimes”, “often”, or “always” said untrue things to avoid being embarrassed.
