# Supplementary material for: Health selection on self-rated health and the healthy migrant effect: Baseline and 1-year results from the health of Philippine Emigrants Study
Source: PLOS Glob Public Health. 2022 Jul 22;2(7):e0000324. doi: 10.1371/journal.pgph.0000324 (PMC9450558; doi:10.1371/journal.pgph.0000324)
Supplement: Attachment — Submitted filename: PGPH-D-21-00506 Response to Reviewer Comments - 220310.docx [file pgph.0000324.s007.docx]

**PGPH-D-21-00506 Response to Reviewer Comments**

**Authors’ General Response to Reviewer Comments**

Thank you for reviewing our paper, entitled “Health Selection on Self-Rated Health and the Healthy Migrant Effect: Baseline and 1-Year Results from the Health of Philippine Emigrants Study”. The reviewers’ comments primarily concerned the significance of examining Filipinos as the study population and more clarification on methodological choices. Our paper has been made stronger with your critical feedback. We have provided responses to each comment (bolded) point-by-point below.

**Editor’s Comments/Journal Requirements**

1. **Please amend your detailed Financial Disclosure statement. This is published with the article, therefore should be completed in full sentences and contain the exact wording you wish to be published. Please state what role the funders took in the study. If the funders had no role in your study, please state: “The funders had no role in study design, data collection and analysis, decision to publish, or preparation of the manuscript.**”

Done.

2. **Please update your Competing Interests statement. If you have no competing interests to declare, please state: “The authors have declared that no competing interests exist.”**

Done.

3. **In the online submission form, you indicated that “The data underlying this article will be shared on reasonable request to the corresponding author.”. All PLOS journals now require all data underlying the findings described in their manuscript to be freely available to other researchers, either 1. In a public repository, 2. Within the manuscript itself, or 3. Uploaded as supplementary information. This policy applies to all data except where public deposition would breach compliance with the protocol approved by your research ethics board. If your data cannot be made publicly available for ethical or legal reasons (e.g., public availability would compromise patient privacy), please explain your reasons by return email and your exemption request will be escalated to the editor for approval. Your exemption request will be handled independently and will not hold up the peer review process, but will need to be resolved should your manuscript be accepted for publication. One of the Editorial team will then be in touch if there are any issues.**

The Health of Philippine Emigrants Study (HoPES) data are available to researchers via a “Data Use Agreement”, which helps to protect the identities of participants and ensures data integrity. Our entire migrant sample consists of newly arrived immigrants. Immigration is a contentious issue within the United States and there have been some political entities that have sought to revoke visas for immigrants. Therefore, our data are accessible, but protected via a screening process to ensure that users are seeking the data for research purposes only. We have provided the aforementioned language in the “Data Availability” statement.

**4. We notice that your supplementary tables are included in the manuscript file. Please remove them and upload them with the file type 'Supporting Information'. Please ensure that all Supporting Information files are included correctly and that each one has a legend listed in the manuscript after the references list.**

Done.

**5. Please review your reference list to ensure that it is complete and correct. If you have cited papers that have been retracted, please include the rationale for doing so in the manuscript text, or remove these references and replace them with relevant current references. Any changes to the reference list should be mentioned in the rebuttal letter that accompanies your revised manuscript. If you need to cite a retracted article, indicate the article’s retracted status in the References list and also include a citation and full reference for the retraction notice.**

We have reviewed the reference list to ensure that it is complete and correct. We have included additional references in response to the reviewers’ request to expand additional sections.

**Reviewer #1 Thank you for the opportunity of reading this interesting manuscript. The study is original and relevant, it is focused on one particular migrant group (emigrant Philippines in the US) but provides wider insights on the health selection model. I think the manuscript is well-written and adequate for publication, but could be improved for including the following comments/corrections:**
**1. In intro, when mentioning allostatic load concept, it should be defined and referrenced.**

In addition to the definition of allostatic load we provide in the “Methods”, we have provided a definition for allostatic load based on McEwen (1998).

**2. In intro, page 6 the paragraph that reads "Given the abundance of mixed evidence..." I actually found only 5 papers related to this, I would not acll it abundant... Please specify more clearly and consistent with existing data.**

We have clarified this language and included additional studies to highlight the inconsistency in mixed evidence regarding health selection on page 7.

**3. In intro, page 8, Filipino migrants are described as a unique type of migrant, how do they compare to other migrants relevant to the US and the world... Could you better justify this, in order to improve the relevance of the paper?**

We have provided further discussion about the uniqueness of migration from the Philippines by highlighting how Filipinos comprise one of the four major immigrant streams to the U.S. (along with China, India, and Mexico), beginning on page 7. We also highlight their unique relationship to the U.S as a former colonial holding as the result of the Spanish-American and Philippine-American wars. Finally, we provide some discussion on the health status of Filipinos compared to other Asian and immigrant groups, noting how despite having better socioeconomic outcomes that should preclude worse health, Filipinos continue to have worse health relative to other groups.

**4. In intro page 9 right before methods, you describe 2 hypothesis, which seem correct but assume independence between dimensions or variables... I am a bit worried about this, as they are interconnected. Could you better justify this, as well as your general OLS approach versus other techniques like SEM in relation to your objectives?**

Our goal is to examine the relative contributions of factors including physical health, mental health, social capital, and socioeconomic status to explaining differences in SRH between migrants and non-migrants. OLS is well-suited to this objective, since it specifically quantifies the additional contribution of independent variables to explaining variation in the dependent variable, beyond that variation explained by other variables. We acknowledge that SEM might be chosen for examining research questions similar to ours. However, since we were not interested exploring relationships among constructs and potential mediation pathways, we did not select SEM as our tool. OLS provides a more parsimonious approach. Nevertheless, we acknowledge that SEM would be an alternative approach and we have included some additional language in the “Discussion” to acknowledge the advantages of SEM, and opportunities to move forward.

**5. Methods: the study groups are people who legally migrated from the Philippes. Is there undocumented Filippino migration to the US? how large is it? How this study would/not inform abiout them?**

Only 3% of Filipinos in the U.S. are undocumented (1). While their experiences are certainly important, our study does not provide information about their experiences. We have added a note in the discussion that acknowledges that our study omits this group.

**6. Age range of study participants 20-59, why? many migrants, as explained in the paper, come for reunification and could be older...**

HoPES is a longitudinal study that focuses on how immigrants’ well-being changes with migration. Part of this goal was to examine employment trajectories. Therefore, we included persons who were in their prime working ages and who might later enter into retirement in later waves of the study.

**7. Methods please better specify how stratified random sampling of non-migrant people was conducted. Why these cities only? do most migrants of the study came from these cities? (you only list refs 24.25, please give some more detail)**

We have provided further detail on the techniques of stratified random sampling of the non-migrant cohort on page 12. Briefly, Cebu and Manila were chosen because those were the two major sites of the Pre-Departure Orientation Seminar (PDOS) offered by the Philippines Government at the time the study was initiated. In order to take the PDOS, migrants from all over the Philippines had to travel to either Cebu or Manila. We acknowledge that the samples are not fully comparable with regards to geographic origin in the Discussion.

**8. Methids many variables are instruments, no discussion around intercultural adequacy for Filippinos was described, please add. How stable are these measures over time in similar populations?**

Each scale used in HoPES has been tested among Filipinos or intended for use among multicultural samples. We have provided additional clarification for each scale on its appropriateness and use within the Filipino population. Importantly, the present analyses employ the same scales for both the migrants and non-migrants. We have no reason to suspect differential bias among instruments between the 2 cohorts. We actually expect that the scores on these instruments to change for the migrants due to the effect of migration. For non-migrants, many estimates appear relatively stable (e.g. high social desirability score is 20.6% at baseline and 19.7% at year 1).

**9. The healthy diet variable seems really rough, please justify.**

We have provided additional justification for using the Food Frequency Questionnaire, highlighting its previous use in the pilot study for HoPES and as behavioral predictors of obesity, a commonly used indicator of overall health. We chose these four diet behaviors, as increased fruit and vegetable consumption is linked to cancer and cardiovascular risk (2) while fast food and soda consumption are linked to cardiovascular risk (3).

**10. In socioeconomic factors you added healthcare utilization, which is often treated separately. Why? Would it not potentially affect self-rated health of migrants over time (beyond first year....)**

We edited this version to not claim that healthcare utilization is a socioeconomic factor. Rather, we just call it what it is (i.e. healthcare utilization).	The 1-year follow up wave of HoPES did not include any questions on healthcare utilization. Thus, we were unable to include a time specific version of the construct for this study.

**11. Discussion: in the best model, 44% of variace is explained by the model. This is highlighted in the paper, but little is discussed around the other 66% that needs further explanation. Please expand.**

We have reworded our discussion to include additional components that could comprise the unexplained 66% of the variance in self-rated health. These components include: cultural and historical factors, and positive affect

**12. Discussion mentions the "health in all policies approach" but it is very poorly explained and developed. Please expand in light of your findings.**

Upon review of our revised paper, we felt that our discussion of the “Health in All Policies” approach felt out of place given our focus of the paper on examining the migrant health advantage and possible explanatory factors. We have chosen to remove this discussion from the conclusion.

**Reviewer #2** Self-rated health (SRH) is an important subject for consideration in migration and health research. The authors of this manuscript have authoritatively stated this in the Introduction. Interestingly, the authors have pointed out that there are factors as determinants responsible for this situation that needs to be clearly defined and their dynamics understood. Thus, the study is set to examine differences in self-rated health between migrants to the U.S. and a comparable group of non-migrants at baseline (premigration) and one year later, accounting for seven domains: physical health, mental health, health behavior, demographics, socioeconomic factors, psychosocial factors, and social desirability. This study design would contribute to the existing literature on migration and health as it allows us to compare migrants to those who did not migrate in the country of origin (i.e. non-migrants). It also allows us to examine health selection at two time points: pre-migration and 1-year post-migration.
In the context of social determinants of (migration) health which the study seems to refer, this reviewer has these observations and comments;

**1. With this as context, the reviewer suggests an inclusion of social determinants of (migration) health frame in the literature review. This would make the introduction clearer and connect well with the study.**
 **- add a paragraph about SDoH, think about how it relates to migration.**

We have included a brief discussion of the SDoH, specifically the role of immigration on health and health selection on Page 4.

**2. The absence of a review of theoretical constructs of social determinants of health and how this links with health and impact on migration has weakened the conceptual grounding of the study.**

On page 4, we provide a brief overview of the social determinants of health framework by identifying some aspects of the framework. We then cite Casteneda et al. (2015) relating social determinants of health to immigration. Finally, we introduce how social determinants of health, as a framework that encompasses multiple domains can be related to self-rated health, a measure of health that encompasses multiple domains. We conclude this opening paragraph by focusing on health selection, which has been a point of major exploration in immigration research.

**3. Methodological approach would be strengthened by stating the study design first, followed by a statement showing, in clear terms as the convention is, that the study is part of a national study.**

We have clarified the language of the study design of HoPES in general, in addition to the current study in the “Data” section under “Materials and Methods”. Per our discussion, HoPES was not nationally representative of all Filipinos in the Philippines. Instead, it is intended to be representative of recent Filipino migrants to the U.S., as indicated by Gee, de Castro (5).

**4. The statistical analysis appears to be properly done and interpretation of the results is correct. An improvement could be done on being consistent with the use of conventional statistical symbols (b, B and beta).**

For consistency, we have used the beta symbol (β) for all discussions of regression coefficients.

**5. Discussion of the results could be more structured to highlight more on the limitations of the methodology employed considering that this was only at two time points.**

We have provided further discussion of the limitations of this analysis of two time points in our “Limitations” section. We highlight the uncertainty of using only two time points to declare that a clear migrant advantage persists. We note how future analyses should incorporate more waves of HoPES as they become available while balancing issues that occur with further attrition.

**Overall, this is an interesting paper to read.**

Thank you.

**References**

1.	Migration Policy Institute. Filipino Immigrants in the United States Washington D.C., USA: Migration Policy Institute; 2020 [Available from: <https://www.migrationpolicy.org/article/filipino-immigrants-united-states-2020#Immigration_Pathways_and_Naturalization>

2.	Boeing H, Bechthold A, Bub A, Ellinger S, Haller D, Kroke A, et al. Critical review: vegetables and fruit in the prevention of chronic diseases. Eur J Nutr. 2012;51(6):637-63.

3.	Hu FB. Globalization of Food Patterns and Cardiovascular Disease Risk. Circulation. 2008;118(19):1913-4.

4.	Castañeda H, Holmes SM, Madrigal DS, Young M-ED, Beyeler N, Quesada J. Immigration as a Social Determinant of Health. Annual Review of Public Health. 2015;36(1):375-92.

5.	Gee GC, de Castro A, Crespi CM, Wang MC, Llave K, Brindle E, et al. Health of Philippine Emigrants Study (HoPES): study design and rationale. BMC public health. 2018;18(1):771.
