# Supplementary material for: Health selection on self-rated health and the healthy migrant effect: Baseline and 1-year results from the health of Philippine Emigrants Study
Source: PLOS Glob Public Health. 2022 Jul 22;2(7):e0000324. doi: 10.1371/journal.pgph.0000324 (PMC9450558; doi:10.1371/journal.pgph.0000324)
Supplement: Attachment — Submitted filename: 220525 - PGPH-D-21-00506-R1 Response to Reviewer Comments.docx [file pgph.0000324.s008.docx]

**PGPH-D-21-00506 Response to Reviewer Comments:**

Thank you for your feedback regarding our paper. We have provided our responses to each comment, point-by-point, below.

**Journal Requirements:

Please review your reference list to ensure that it is complete and correct. If you have cited papers that have been retracted, please include the rationale for doing so in the manuscript text, or remove these references and replace them with relevant current references. Any changes to the reference list should be mentioned in the rebuttal letter that accompanies your revised manuscript. If you need to cite a retracted article, indicate the article’s retracted status in the References list and also include a citation and full reference for the retraction notice.**

We have reviewed the reference list and included the following citations:

1. Kreuter, Frauke, and Richard Valliant. 2007. "A Survey on Survey Statistics: What is Done and Can be Done in Stata." *The Stata Journal* 7(1):1-21.
2. Lubbers, M., and M. Gijsberts. 2019. "Changes in Self-Rated Health Right After Immigration: A Panel Study of Economic, Social, Cultural, and Emotional Explanations of Self-Rated Health Among Immigrants in the Netherlands." *Front Sociol* 4:45.
3. StataCorp, LP. 1985. "Stata survey data reference manual." *Citeseer*.
4. Williams, Richard. 2006. "Generalized Ordered Logit/Partial Proportional Odds Models for Ordinal Dependent Variables." *The Stata Journal* 6(1):58-8
5. Williams, Richard. 2016. "Understanding and interpreting generalized ordered logit models." *The Journal of Mathematical Sociology* 40(1):7-20.
6. Wolfe, Rory. 1997. "OMODEL: Stata modules to perform tests on orderer probit and ordered logit models.

None of the papers included are retracted papers. **Additional Editor Comments (if provided):

Thank you for addressing the comments made by the reviewers. However, there remains some technical issues that needs to be clarified before this paper can be recommended for publication.

1. First, the measure of SRH was regarded continuous in the analyses. The results of the OLS (in Table 2) suggest that the expected level of SRH among migrants is 0.77 unit higher than that among non-migrants. However, the SRH status is subjective; and movement from poor to fair (i.e., 0 to 1) and very good to excellent (i.e., 3 to 4) though by 1 units in both cases but very different in subjective measure. Therefore, it warrants solid rationale why a continuous measure of SRH is appropriate to answer the question asked in this paper. Please justify the use of continuous SRH measure as the outcome variable and adequately discuss the statistical issues related to it. Also please adequately discuss the interpretation of a continuous SRH in the context of the research question, and clearly outline any limitations associated with the choice of outcome variable.**

The primary goal of our study was to examine if migrants had better SRH compared to non-migrants both before and 1-year after migration. The secondary goal was to quantify the extent to which demographic, social, socioeconomic, and health factors accounted for differences in SRH by migrant status. Treating SRH as a continuous outcome provided us with coefficients that are easier to interpret in addition to the adjusted R-squared, which measures how much of the variance in the outcome may be accounted for by variables included in the model. Our sensitivity analyses examining a dichotomous and ordinal measure of SRH supported the conclusions from our continuous analyses. We have provided justification for our choice in our “Methods” section on page 13 and 14:

“We chose to examine SRH as a continuous variable because it provides easily interpretable coefficients and adjusted R-squared measures. The use of adjusted R-squared allows us to quantify how much variance in SRH may be accounted for by variables in the model. By looking at nested models, we can further evaluate how much the adjusted R-squared increases with inclusion of blocks of variables.

We supplement our analyses of SRH as a continuous variable by conducting sensitivity analyses examining SRH as a binary outcome (poor/fair vs. good/very good/excellent health) and 5-level ordinal outcome”

The sensitivity analyses showed that our results produced when treating SRH as a binary outcome and as an ordinal outcome were similar to our main results treating SRH as a continuous outcome (see page 22).

We have noted the limitations that you have mentioned in interpreting SRH as a continuous outcome in the “Discussion” section on page 27:

“Furthermore, using OLS may have additional statistical issues related to qualitative interpretation of the coefficients. For example, a 1-unit change from “poor” to “fair” health may be qualitatively different than a one-unit change from “very good” to “excellent health”. It is also possible that a one-unit change may also be qualitatively different by migrant status. A panel study of recent immigrants to the Netherlands found that more immigrants rated their health as “good” rather than “very good” only a few years post-migration (1). The authors concluded that although immigrants continued to maintain “good” health upon their migration, unmet expectations of immigration, greater involvement in hazardous work, homesickness, and discrimination from Dutch inhabitants contributed to this downward transition. However, we show in our supplemental analyses that trends between migrants and non-migrants remained the same, even after accounting for possible social and psychological factors. Migrants continued to have a health advantage and report better SRH compared to non-migrants regardless of operationalization of SRH. Ultimately, our use of OLS provides a starting point for future research to examine the effects of these factors and additional health and social factors in future waves of HoPES.”

**2. Second, one of the necessary criteria of ordinal logistic regression in fulfillment of the proportional odds assumption. If that assumption does not hold, then the estimates of a ordinal/ordered logistic regression is not valid. I do not see any mention of the proportional odds assumption in the manuscript, satisfying which is a prerequisite of carrying out an ordinal logistic specification. Please conduct appropriate statistical checks to confirm whether the proportional odds assumption was duly satisfied. If yes, clearly mention that in the manuscript. If no, then replace the ordinal logistic analysis with multinomial logistic model and report the results accordingly.**

We used the “omodel” Stata user command to test the proportional odds assumption for the ordinal logistic regression models. The test is performed by comparing the fit of the ordinal logistic regression model (with proportional odds assumption) to a generalized ordered logit model (more flexible parametrization that does not assume proportional odds) using a likelihood ratio test. We found that the proportional odds assumption was not violated for our key variable of interest, migrant status, in the crude model (Likelihood-Ratio Test: X^2^ (3 df) = 4.65, p = .200). In the full model, likelihood ratio test indicated that the generalized ordered logit model provided a better fit (Likelihood-Ratio Test: X^2^ (58 df) = 92.16, p = .003), suggesting that the proportional odds assumption may have been violated for one or more other variables in the model.

We then used the “gologit2” Stata user command, which allows the user to fit partial proportional odds models in which some covariates have the proportional odds assumption and some not, to examine if migrant status violated the proportional odds assumption in the full generalized logit model. We found that migrant status did not violate the assumption (p = .462).

Given that the proportional odds assumption was not violated for our key variable of interest, that the ordinal logistic regression approach was conducted as a sensitivity analysis rather than the main analysis, and that the generalized logit model had many more parameters and was difficult to interpret, we chose to retain the ordinal logistic regression approach. We provide our reasoning for our decision on page 18:

“In our sensitivity analyses of SRH as a 5-level ordinal outcome, we evaluated whether the proportional odds assumption was met using the “omodel” user written command in Stata (2). We found that the proportional odds assumption was not violated for our key variable of interest, migrant status, in a crude bivariate model (Likelihood-Ratio Test of Proportionality of Odds: X^2^ (3 df) = 4.65, p = .200). However, a global test indicated that the proportional odds assumption was violated for one or more variables in the fully adjusted model (Likelihood-Ratio Test of Proportionality of Odds: X^2^ (58 df) = 92.16, p = .003). Further exploration using the “gologit2” user written command in Stata (3) revealed that migrant status was not one of the variables that violated the proportional odds assumption in the fully adjusted model (p = .462). For this reason and for parsimony, we chose to retain the ordinal logistic regression, instead of exploring other generalized models for our sensitivity analyses (3, 4).”

**3. It is not explicitly mentioned how standard errors were estimated in the OLS and the logistic regression framework. I guess, the authors assumed homoskedasticity as I did not see any mention of robust standard errors. Homoscedasticity or constant variance of the error term is rare in real life data, and it is convention to provide robust standard error estimates. Please clearly state how the standard errors were estimated in the regression. If it is not robust, provide evidence of homoscedasticity. If the errors are heteroskedastic, then provide appropriate standard errors (or confidence intervals) that are robust to heteroskedasticity.**

We used the “svyset” command in Stata to apply post-stratification weights (for baseline) and propensity weights (for 1-year) to the HoPES dataset. These weights account for the complex survey design of HoPES (for baseline) and the attrition experienced in the 1-year sample. In all of our multivariable analyses, we used the “svy” command, which produces robust standard errors that account for the complexity of the study design in both OLS and logistic models. We have provided further clarification of the survey weights on pages 17 and 18 as follows:

“Both post-stratification weights and propensity weights were applied to the dataset using the “svyset” command in Stata (5, 6).

We first examined differences in each domain by migrant status using t-tests and chi-square tests at both baseline and 1-year. We then fit a series of ordinary least squares (OLS) regression models adding each domain in the following order: migrant status, demographics, physical health, mental health, health behaviors, socioeconomic status and healthcare utilization, psychosocial factors, and social desirability using the “svy” command. The “svy” command produces robust standard errors that account for the complexity of the study design.”

1.	Lubbers M, Gijsberts M. Changes in Self-Rated Health Right After Immigration: A Panel Study of Economic, Social, Cultural, and Emotional Explanations of Self-Rated Health Among Immigrants in the Netherlands. Front Sociol. 2019;4:45.

2.	Wolfe R. OMODEL: Stata modules to perform tests on ordered probit and ordered logit models. 1997.

3.	Williams R. Generalized Ordered Logit/Partial Proportional Odds Models for Ordinal Dependent Variables. The Stata Journal. 2006;6(1):58-82.

4.	Williams R. Understanding and interpreting generalized ordered logit models. The Journal of Mathematical Sociology. 2016;40(1):7-20.

5.	Kreuter F, Valliant R. A Survey on Survey Statistics: What is Done and Can be Done in Stata. The Stata Journal. 2007;7(1):1-21.

6.	StataCorp L. Stata survey data reference manual. Citeseer. 1985.
